# Liberalization of the Systemic Glucose Management is Associated with a Reduced Frequency of Neuroglucopenia in Subarachnoid Hemorrhage Patients: An Observational Cohort Study

**DOI:** 10.1007/s12028-024-02126-8

**Published:** 2024-10-15

**Authors:** Mario Kofler, Anna Lindner, Verena Rass, Bogdan A. Ianosi, Lauma Putnina, Philipp Kindl, Alois J. Schiefecker, Maxime Gaasch, Ronny Beer, Paul Rhomberg, Claudius Thomé, Erich Schmutzhard, Bettina Pfausler, Raimund Helbok

**Affiliations:** 1 Department of Anaesthesiology and Critical Care Medicine, Medical University of Innsbruck, Innsbruck, Austria; 2 Department of Neurology, Medical University of Innsbruck, Innsbruck, Austria; 3 Department of Medical Informatics, University for Health Sciences, Medical Informatics, and Technology, Hall in Tirol, Austria; 4 Department of Neuroradiology, Medical University of Innsbruck, Innsbruck, Austria; 5 Department of Neurosurgery, Medical University of Innsbruck, Innsbruck, Austria; 6 Department of Neurology, Kepler University Hospital, Johannes Kepler University Linz, Linz, Austria; 7 Clinical Research Institute of Neuroscience, Johannes Kepler University Linz, Kepler University Hospital, Linz, Austria

**Keywords:** Subarachnoid hemorrhage, Cerebral microdialysis, Cerebral metabolism, Glucose, Neurocritical care

## Abstract

**Background:**

Pathologically low brain glucose levels, referred to as neuroglucopenia, are associated with unfavorable outcomes in neurocritical care patients. We sought to investigate whether an increase in serum glucose levels would be associated with a reduction of neuroglucopenia.

**Methods:**

In this retrospective analysis of prospectively collected data, we included 55 consecutive patients with spontaneous subarachnoid hemorrhage who underwent cerebral microdialysis (CMD) monitoring. Neuroglucopenia was defined as CMD-glucose levels < 0.7 mmol/l. We identified systemic glucose liberalization events, defined as a day with median serum glucose levels < 150 mg/dl, followed by a day with median serum glucose levels > 150 mg/dl, and compared concentrations of cerebral metabolites between these days. Unfavorable outcome was defined as modified Rankin Scale score ≥ 3 at 3 months after the bleeding.

**Results:**

Episodes of neuroglucopenia were more frequent in patients with unfavorable outcome (19.8% [19.3–20.3%] vs. 10.9% [10.4–11.5%], *p* = 0.007). Sixty-nine systemic glucose liberalization events were identified in 40 patients. Blood glucose levels increased from 141.2 (138.7–143.6) mg/dl to 159.5 (157.0–162.2) mg/dl (*p* < 0.001), CMD-glucose levels increased from 1.44 (1.39–1.50) mmol/l to 1.68 (1.62–1.75) mmol/l (*p* = 0.001), and the frequency of neuroglucopenia decreased from 24.7% (22.9–26.5%) to 20.2% (18.7–21.8%) (*p* = 0.002) during these events. Liberalization was not associated with changes in CMD-lactate, CMD-pyruvate, CMD–lactate-to-pyruvate ratio, CMD-glutamate, or CMD-glycerol.

**Conclusions:**

In conclusion, the liberalization of serum glucose concentrations to levels between 150 and 180 mg/dl was associated with a significant reduction of neuroglucopenia.

**Supplementary Information:**

The online version contains supplementary material available at 10.1007/s12028-024-02126-8.

## Introduction

Despite treatment advances, subarachnoid hemorrhage (SAH) remains a devastating disease, with high rates of mortality, morbidity, permanent disability, and neuropsychological deficits [1, 2]. Brain metabolic derangement has been found to be an important contributing factor to the complex pathophysiology of early and secondary brain injury following SAH [3]. The impairment of cerebral metabolism can be monitored using cerebral microdialysis (CMD).

Glucose is the primary substrate for cerebral energy production. Brain glucose levels depend on systemic delivery, transportation across the blood–brain barrier, and cerebral glucose consumption. Pathologically low brain glucose concentrations, referred to as neuroglucopenia, have been associated with increased mortality, unfavorable functional outcome, and delayed cerebral ischemia (DCI) in patients with SAH [3]. Accordingly, an expert panel suggested the treatment of pathologically low brain glucose levels [4]. The administration of enteral nutrition has already been shown to increase CMD-glucose levels [5].

The optimal range of serum glucose levels in critically ill patients remains a matter of debate [6]. In neurological intensive care patients, there is some evidence that a more liberal systemic glucose management protocol may be advantageous compared with tight glycemic control (80–110 mg/dl or 80–120 mg/dl), as the former was associated with less episodes of cerebral metabolic distress and neuroglucopenia [7]. Furthermore, insulin appears to have a more pronounced effect on cerebral glucose concentrations than on serum glucose levels [8, 9].

We hypothesized that the liberalization of the systemic glucose management, in terms of accepting higher serum glucose concentrations, would lead to an increase in brain glucose levels and a reduction of the frequency of neuroglucopenia.

## Material and Methods

This is a retrospective analysis of data collected in an ongoing prospective observational cohort study on consecutive patients with spontaneous SAH and cerebral microdialysis admitted to the neurological intensive care unit of a tertiary referral center between 2010 and 2016. The article was written according to the Strengthening the Reporting of Observational studies in Epidemiology guidelines. All provisions of the Helsinki Declaration were followed. The conduct of this study was approved by the ethics committee of the Medical University of Innsbruck, Austria (AN3898 285/4.8), and informed consent was obtained from all patients or a legal representative, according to Austrian legislation. Inclusion criteria were age above 18 years, the admission diagnosis of spontaneous SAH, and cerebral microdialysis monitoring for at least 48 h.

### Treatment and Grading

The treatment of patients conformed to current international guidelines, with the exception of nimodipine being administered intravenously [10, 11]. Ruptured aneurysms were treated early, either by endovascular coiling or neurosurgical clipping. If no aneurysm was detected, a repeat conventional angiography was performed 2 weeks after admission. All patients were mechanically ventilated during the period of invasive neuromonitoring and routinely received continuous midazolam and sufentanil. In case of hydrocephalus, an external ventricular drain was placed. If intracranial hypertension (> 20 mm Hg for > 5 min) was detected, despite routinely performed preventive measures (head and upper body positioning, adequate sedation, normoventilation, normothermia), possible interventions included deeper sedation, mild hyperventilation, osmotherapy, and, in refractory cases, hemicraniectomy. All patients were monitored by transcranial color-coded duplex sonography. When severe vasospasm (> 200 cm/s) was detected, induced hypertension with a target cerebral perfusion pressure (CPP) > 80 mm Hg was applied, and intraarterial nimodipine administration was considered. DCI was defined as a new infarct detected by cerebral computed tomography (CT) or magnetic resonance imaging scanning, not attributable to other causes [12]. Functional outcome was prospectively assessed 3 months after intensive care unit admission by a telephone interview, performed by a trained study nurse masked to clinical data, using the modified Rankin Scale. Scores of 0–2 were defined as favorable outcomes, and scores of 3–6 were defined as unfavorable functional outcomes.

Clinical disease severity was graded using the Hunt and Hess scale. Cerebral CT was performed on admission, after aneurysm treatment, and whenever clinically indicated. The initial CT scans were graded by a neuroradiologist using the modified Fisher score and screened for the presence of global cerebral edema [13, 14]. Microdialysis probe location was defined as “perilesional” if the gold tip of the probe was within 1 cm of a focal CT hyperdense or hypodense lesion or, if not, as “normal-appearing brain tissue” [15].

### Cerebral Microdialysis

Invasive neuromonitoring, including cerebral microdialysis, was performed in patients who were expected to be mechanically ventilated for more than 48 h and showed clinical and/or radiologic signs indicative of raised intracranial pressure as part of clinical routine monitoring. CMD catheters (71 High Cut-Off Brain Microdialysis Catheter; M Dialysis AB, Stockholm, Sweden) were tunneled and placed into the white matter of the brain region deemed to be at the highest risk of developing secondary brain injury (the vascular territory of the ruptured aneurysm or the region exhibiting the severest focal brain lesion). Isotonic perfusion fluid (Perfusion Fluid CNS; M Dialysis AB) was pumped through the microdialysis system at a flow rate of 0.3 µl/min. Hourly samples were immediately analyzed with a CMA 600 or Iscus^flex^ (M Dialysis AB) for CMD-glucose, CMD-pyruvate, CMD-lactate, and CMD-glutamate concentrations. At least 1 h passed between the insertion of the probe and the start of sampling. Neuroglucopenia was defined as CMD-glucose < 0.7 mmol/l, metabolic distress was defined as lactate-to-pyruvate ratio (LPR) > 40 and mitochondrial dysfunction as LPR > 30 together with pyruvate > 70 µmol/l [3].

### Glycemic Control and Liberalization

Our institutional protocol aims at maintaining serum glucose concentrations between 110 and 180 mg/dl (6.1–10 mmol/l). Continuous short-acting intravenous insulin is administered if serum glucose concentrations exceed this threshold. Within this range, the determination of a patient’s individual serum glucose target is left to the discretion of the treating physician, also considering CMD-glucose levels. To investigate the impact of systemic glucose on brain glucose levels, we defined a liberalization event as a day with a median serum glucose concentration < 150 mg/dl (8.3 mmol/l) followed by a day with a median serum glucose concentration > 150 mg/dl (8.3 mmol/l) and compared variables (especially the frequency of neuroglucopenia) between these consecutive days. This way, we sought to investigate the impact of changes of serum glucose levels from the “lower normal range” (i.e., 110–150 mg/dl) to the “higher normal range” (150–180 mg/dl) on cerebral physiology within individuals on directly consecutive time frames. Hyperglycemia was defined as serum glucose > 200 mg/dl.

### Statistics

Numeric data are shown as counts and percentages per group. Continuous data were assessed for normality using the Shapiro–Wilk test and are shown as means and 95% confidence intervals or as medians and interquartile ranges. Repeated measurements within study participants were performed using generalized estimating equations, choosing the regression matrix with the best fit for the data. Cases with missing values were included. For the assessment of temporal dynamics, “day after the hemorrhage” was inserted as a factor in a linear model, with CMD-glucose (single values) or percentage of daily neuroglucopenia (percentage of measurements per day per patient) as an outcome variable. In a similar manner, the impact of liberalization was analyzed by inserting the binominal “before and after liberalization” variable as factor into a linear model. The impact of CMD-glucose on functional outcome was assessed in a binary-logistic model, with the dichotomized modified Rankin Scale as an outcome variable and CMD-glucose (single values) or percentage of neuroglucopenia (percent of measurements per day per patient) as a covariate. The level of significance was set at a *p* value < 0.05. All analyses were performed with SPSS (Version 24.0; IBM SPSS Statistics, Armonk, NY).

## Results

Baseline characteristics, hospital complications and outcome of 55 consecutive patients with SAH with CMD-monitoring are detailed in Table 1. Neuromonitoring data up to 14 days after the hemorrhage are reported. CMD-monitoring was started on day 1 (1–2) after SAH and continued for 9 (6–12) days, overall 8,659 CMD-samples were analyzed. Probe location was perilesional in 34 patients (62%) and in normal-appearing brain tissue in 21 patients (38%).

**Table 1:** Patient characteristics

Characteristics	*n* (%) or median (IQR)
Age	59 (48–67)
Sex (female)	37 (67)
Type 2 diabetes	1 (2)
Hunt & Hess grade (initial)	
2	5 (9)
3	14 (26)
4	6 (11)
5	30 (54)
Loss of conciousness	33 (60)
APACHE II score	18 (15–21)
Modified Fisher grade	
1	5 (9)
2	14 (26)
3	6 (11)
4	30 (54)
SAH sum score	23 (15–27)
IVH sum score	5 (0–10)
Aneurysm size > 10 mm	15 (27)
Global cerebral edema	30 (54)
Hydrocephalus	48 (88)
Clipping	31 (56)
Coiling	23 (42)
Hemicraniectomy	10 (18)
Pneumonia	40 (73)
Sepsis	9 (16)
Delayed cerebral ischemia	14 (25)
Anemia requiring transfusion	30 (54)
Length of ICU stay	38 (25–56)
Modified Rankin scale after 3 months	
0	2 (4)
1	11 (20)
2	3 (6)
3	7 (13)
4	9 (16)
5	15 (27)
6	8 (14)

APACHE = acute physiology and chronic health evaluation; ICU = intensive care unit; IVH = intraventricular hemorrhage; SAH = subarachnoid hemorrhage

**Fig. 1:**
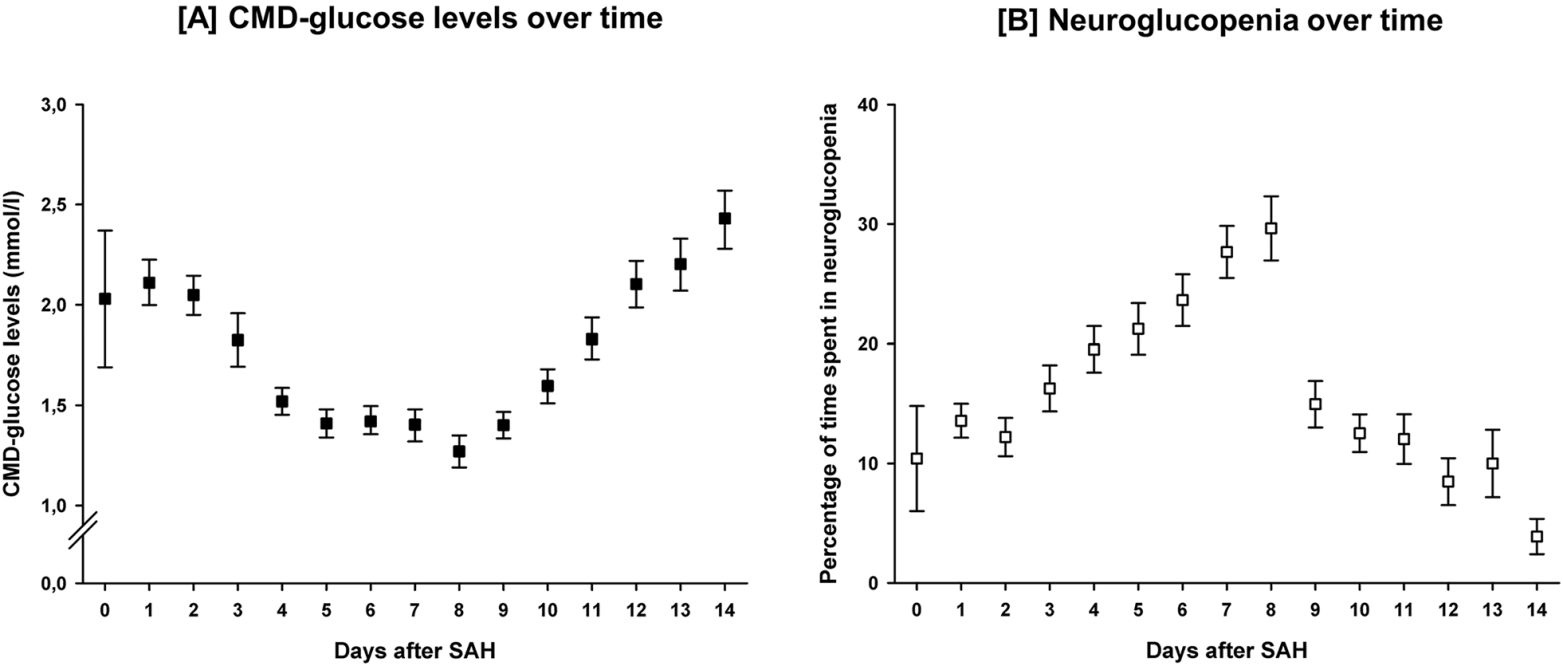
Temporal dynamics of interstitial brain glucose levels (**A**) and the daily frequency of pathologically low brain glucose levels (< 0.7 mmol/l), referred to as neuroglucopenia, (**B**) over 2 weeks after the ictus. Error bars indicate the 95% confidence interval of mean. *N* = 8659 CMD-samples; CMD = cerebral microdialysis; SAH = subarachnoid hemorrhage.

After an initial peak on day one (2.11 mmol/l [1.00–2.23 mmol/l]) CMD-glucose levels decreased to a nadir of 1.27 mmol/l (1.19–1.35 mmol/l) on day 8 (*p* < 0.001) and increased again thereafter (*p* < 0.001). Accordingly, the daily percentage of neuroglucopenia increased from 10.4% (6.0–14.8%) on day 0 to 29.7% (26.9–32.3%) on day 8 (*p* = 0.018) and decreased thereafter (*p* < 0.001). Temporal dynamics of CMD-glucose concentrations and the frequency of neuroglucopenia are shown in Fig. 1. Fifty-one patients (93%) exhibited at least one episode of neuroglucopenia. In patients developing neuroglucopenia, its frequency ranged from 1 to 74% of measurements.

There was no difference in absolute CMD-glucose levels between patients with favorable or unfavorable functional outcome (*p* = 0.98). However, episodes of neuroglucopenia were significantly more frequent in patients with unfavorable compared to favorable functional outcome (19.8% [19.3–20.3%] versus 10.9% [10.4–11.5%], *p* = 0.007). The hazard ratio for unfavorable outcome was 1.015 (95% CI 1.012–1.017) per percent of daily neuroglucopenia. There was a nonsignificant trend toward a higher rate of neuroglucopenia in patients who developed DCI (21.3% [19.9–22.7%] versus 16.5% [15.9–17.1%], *p* = 0.076). Sonography-defined vasospasm was not associated with neuroglucopenia (*p* = 0.249). Furthermore, there was no association between neuroglucopenia and cerebral oxygenation (supplemental appendix). The daily percentage of time spent in neuroglucopenia was significantly higher in patients developing sepsis (27.7% [26.5–28.9%] versus 14.1% [13.4–14.7%], *p* = 0.039), while hyperthermia (central body temperature > 38 °C) was not associated with neuroglucopenia (*p* = 0.623). These analyses were adjusted for age, Hunt & Hess grade, CMD probe location and CPP.

**Fig. 2:**
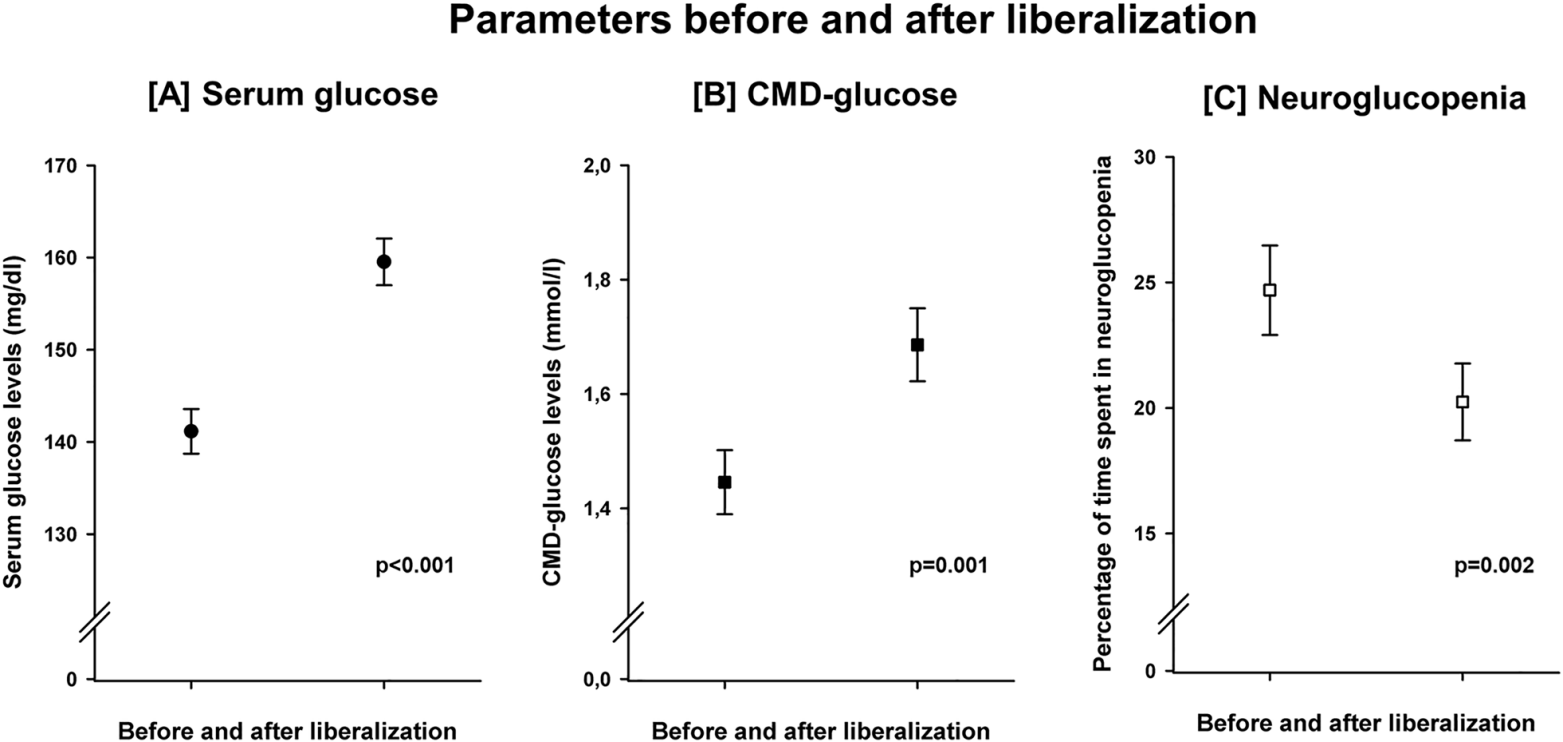
Liberalization was associated with a significant increase of serum glucose levels (**A**, *p* < 0.001) and brain glucose levels (**B**, *p* = 0.001), as well as with a significant reduction of the frequency of pathologically low brain glucose levels (< 0.7 mmol/l), referred to as neuroglucopenia (**C**, *p* = 0.002). Error bars indicate the 95% confidence interval of mean. *N* = 2534 CMD-samples; CMD = cerebral microdialysis.

**Fig. 3:**
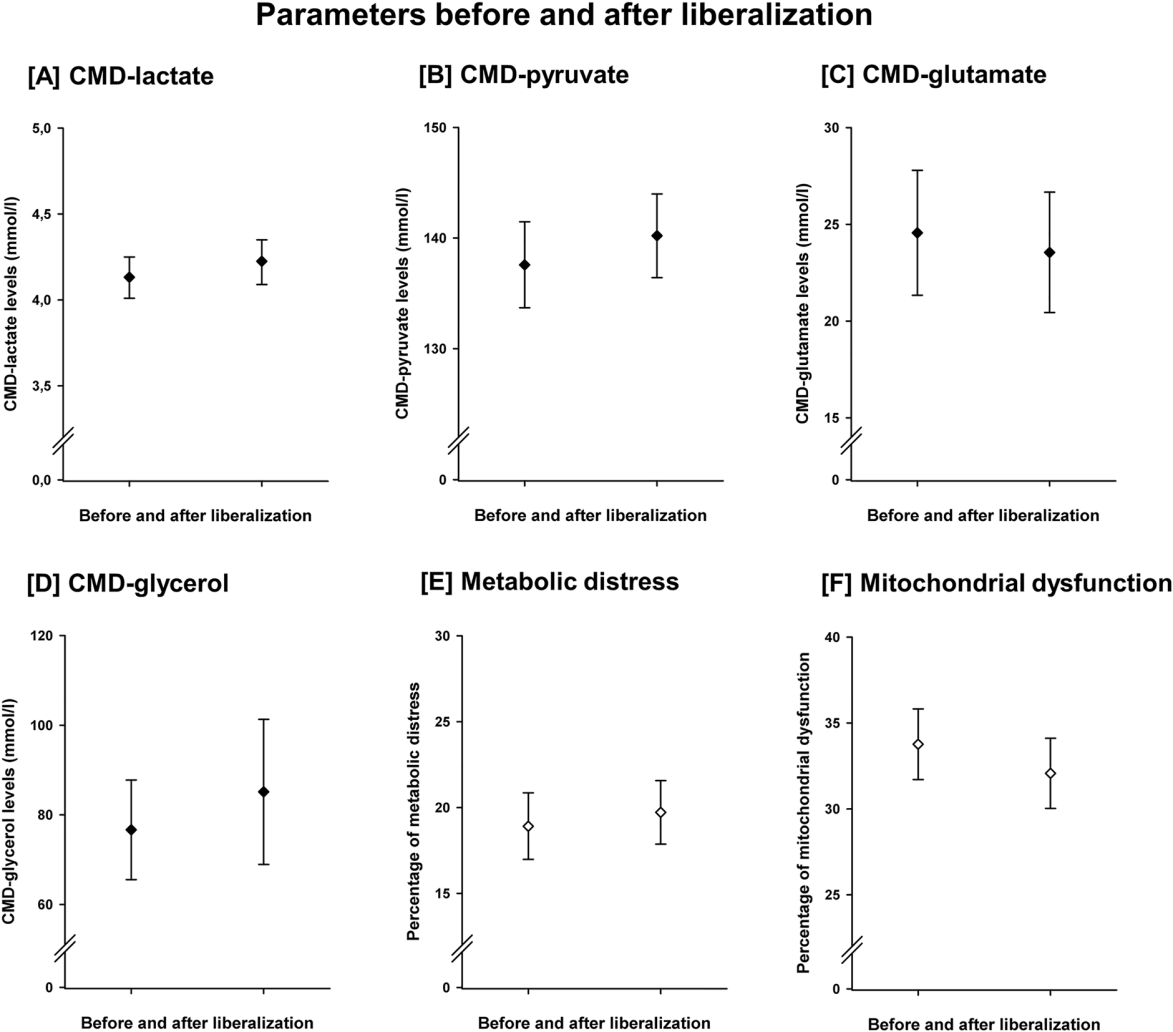
Liberalization was not associated with statistically significant changes of the levels of CMD-lactate (**A**), CMD-pyruvate (**B**), CMD-glutamate (**C**), CMD-glycerol (**D**), the frequency of cerebral metabolic distress (**E**) or the frequency of the cerebral metabolic profile of mitochondrial dysfunction (**F**). Error bars indicate the 95% confidence interval of mean. *N* = 2534 CMD-samples; CMD = cerebral microdialysis.

Sixty-nine systemic glucose liberalization events were identified in 40 patients at a median of 5.5 (4–8) days after SAH. Blood glucose levels increased from 141.2 mg/dl (138.7–143.6 mg/dl) to 159.5 mg/dl (157.0–162.2 mg/dl), *p* < 0.001, CMD-glucose levels increased from 1.44 mmol/l (1.39–1.50 mmol/l) to 1.68 mmol/l (1.62–1.75 mmol/l), *p* = 0.001, and the frequency of neuroglucopenia (per liberalization event) decreased from 24.7% (22.9–26.5%) to 20.2% (18.7–21.8%), *p* = 0.002, during these events, Fig. 2. The frequency of hyperglycemia increased from 3.6 to 6.6% (*p* = 0.023). Hyperglycemia was not associated with functional outcome in this cohort (*p* = 0.783). The insulin dose was reduced from 46.5 U/d (43.3–49.6 U/d) to 41.7 U/d (39.8–43.1 U/d) *p* = 0.033). There was no difference in administered calories by artificial nutrition (1687 vs. 1695 kcal/d, *p* = 0.992). Liberalization events were not associated with changes in CMD-lactate, CMD-pyruvate, CMD-LPR, CMD-glutamate or CMD-glycerol (Fig. 3). Liberalization events were associated with a decrease of the frequency of neuroglucopenia also in perilesional probe location (*n* = 42 liberalization events in *n* = 25 patients, 32.2% [29.7–34.8%] to 23.3% [21.1–25.6%], *p* = 0.011), and during metabolic distress (*n* = 29 liberalization events, 23.6% [21.2–26.0%] to 15.5% [13.6–17.3%], *p* = 0.003). In 44 events, neuroglucopenia occurred at least once on the first day of the liberalization event. In these events, the frequency of neuroglucopenia decreased from 38.1% (35.9–40.4%) to 29.1% (26.9–31.3%), *p* = 0.002.

All analyses were adjusted for age and Hunt & Hess grade, analyses involving CMD-glucose levels were also adjusted for CMD probe location and CPP, except for daily insulin dose, which was not adjusted.

**Fig. 4:**
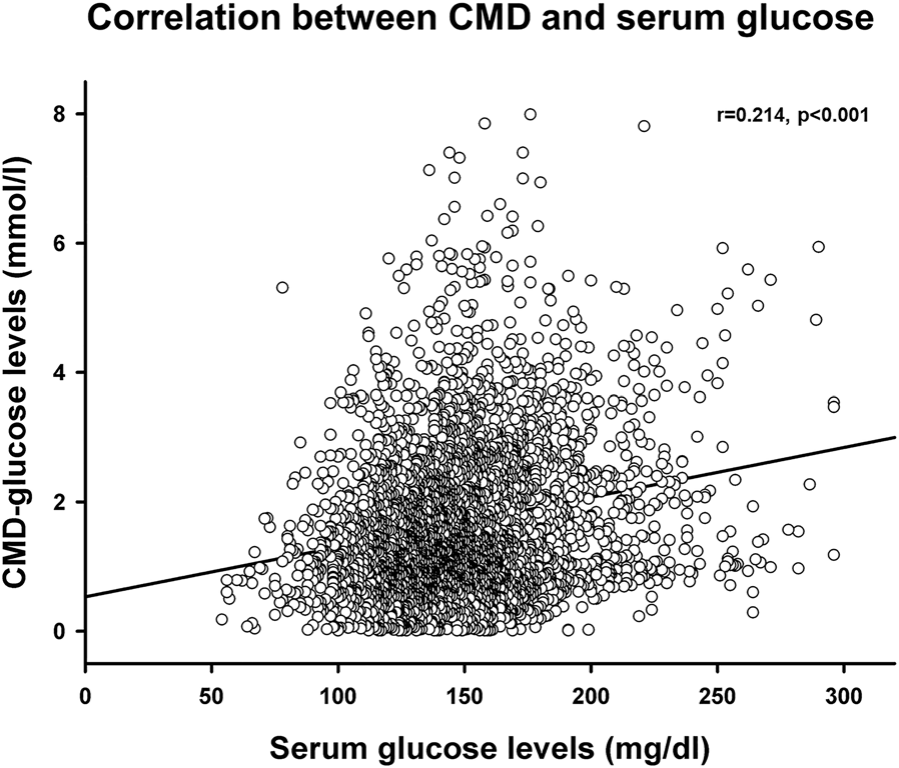
A scatter plot depicting the correlation between serum and CMD-glucose levels. *N* = 3784 pairs of CMD and serum glucose values; CMD = cerebral microdialysis.

There were 3784 pairs of corresponding serum and CMD-glucose values. Overall, there was a weak yet significant correlation between serum glucose and CMD-glucose levels (*r* = 0.214, *p* < 0.001, Fig. 4). The frequency of neuroglucopenia was 30.4% when serum glucose levels were < 110 mg/dl, 19.0% when serum glucose was between 110 and 180 mg/dl and 7.5% when serum glucose levels were > 180 mg/dl.

## Discussion

The main finding of this study is that the liberalization of the systemic glucose management, in terms of accepting higher serum glucose concentrations, is associated with a reduction of the frequency of pathologically low brain glucose levels in patients with SAH. This association was also observed in proximity of focal brain lesions and when cerebral microdialysis parameters indicated metabolic distress.

Neuroglucopenia has been found to be associated with unfavorable functional outcome and DCI in several cohorts of patients with SAH [3]. These findings were replicated in this study (although the association with DCI was not quite statistically significant in the adjusted model). In this regard, an expert consensus statement recommends the treatment of pathologically low brain glucose concentrations [4]. Our data indicate that, besides administering enteral nutrition [5], the liberalization of systemic glucose management is a possible treatment option.

An association between brain and serum glucose levels has been established earlier, however, some studies reported only a weak correlation (as also observed in this cohort) [8, 16]. Especially under conditions of cerebral metabolic distress this relationship appears to be altered [9]. Therefore, it has been questioned whether interventions targeting brain glucose levels could be successful when CMD detects a deranged cerebral metabolism or in the area of focal brain lesions. Our study shows that allowing higher serum glucose levels is associated with a reduced frequency of neuroglucopenia, even if the LPR exceeds 40 or in perilesional brain tissue.

It has been shown that insulin treatment may have a more pronounced impact on brain glucose levels than on serum glucose concentrations, as the frequency of pathologically low brain glucose levels increased after the initiation of insulin treatment, whereas serum glucose levels remained within normal range [8]. The brain also seems to be particularly vulnerable to neuroglucopenia under conditions of metabolic distress at the time of insulin administration [9]. In line with this, more episodes of neuroglucopenia and metabolic distress were found during intensive insulin treatment (target serum glucose 80–120 mg/dl) as compared with a more liberal regimen (121–180 mg/dl) [7]. Our data complement these findings by showing that a minor reduction of the insulin dose is associated with a significant decrease of the frequency of neuroglucopenia. Furthermore, we observed an even lower frequency of neuroglucopenia during serum glucose levels above 180 mg/dl, so that accepting levels beyond this threshold may be an option in patients with refractory neuroglucopenia. It is, however, important to be aware of the potential adverse effects of severe hyperglycemia. In addition, treatable conditions associated with increased glucose consumption (such as sepsis, fever, or seizures) need to be identified.

Whether such an intervention may be beneficial for patients with SAH in terms of improving functional or brain tissue outcome remains elusive. Investigating such an effect is beyond the scope of this observational study. In terms of other cerebral metabolic parameters, neither deterioration nor improvement was detected, similar to earlier findings on the effect of enteral nutrition on brain metabolism [5]. On the other hand, despite remaining low overall, the frequency of hyperglycemia increased significantly with liberalization. Hyperglycemia was associated with unfavorable outcome after SAH and with a more frequent occurrence intensive care unit-acquired weakness in different populations [17, 18]. Therefore, allowing higher serum glucose levels should probably be restricted to patients in whom neuroglucopenia is detected.

Several limitations of our study merit consideration. Besides the inherent limitation of a small sample size in a highly selected population in microdialysis studies of patients with SAH and the small sample volume of brain tissue assessed by CMD (being a local monitoring tool not allowing conclusions regarding global brain metabolism), liberalization events were identified retrospectively and there was neither an explicitly defined threshold that should trigger interventions nor a standardized approach for augmenting serum glucose levels. Because of the retrospective identification, it is not possible to exactly differentiate the active induction of liberalization events from spontaneous increases of serum glucose levels. It is, however, conceivable that many events occurred due to treatment decisions, as neuroglucopenia was present during the larger portion of days before liberalization and the applied insulin dose was lower despite increasing serum glucose levels. The effect of neuroglucopenia on functional outcome may be overestimated or underestimated due to the small sample size and missing values, however, the relationship was highly significant and is in line with earlier studies. In the same context, absolute numbers of CMD-glucose levels and the frequency of neuroglucopenia should be interpreted with caution in this group of highly selected patients. Because other CMD-parameters remained unchanged by liberalization, we cannot conclude that it leads to an improvement of cerebral energy metabolism. This may be due to an already induced state of hyperglycolysis (indicated by the elevated levels of lactate and pyruvate at baseline) and/or increased mitochondrial processing, which is not assessable by cerebral microdialysis. It is important to take into account that this study was not powered to assess the effect of interventions on functional outcome, the results should therefore be perceived as hypothesis-generating insight into cerebral physiology.

## Conclusions

Allowing serum glucose concentrations of 150–180 mg/dl, which we called liberalization of the systemic glucose management protocol, was associated with a reduction of the frequency of neuroglucopenia, a potentially deleterious metabolic condition of the brain. The effect was also observed in perilesional brain tissue and under conditions of brain metabolic distress.

## Supplementary Information

Below is the link to the electronic supplementary material.

Supplementary file1 (PDF 141 kb)
