# Supplementary material for: Liberalization of the Systemic Glucose Management is Associated with a Reduced Frequency of Neuroglucopenia in Subarachnoid Hemorrhage Patients: An Observational Cohort Study
Source: Neurocrit Care. 2024 Oct 15;42(2):343–50. doi: 10.1007/s12028-024-02126-8 (PMC11950053; doi:10.1007/s12028-024-02126-8)
Supplement: Supplementary file 1 — Supplementary file1 (PDF 141 kb) [file 12028_2024_2126_MOESM1_ESM.pdf]

Supplemental appendix

Neuroglucopenia and cerebral oxygenation

Brain tissue oxygen tension (ptiO2) measurements are available in 49 patients. As shown in the supplemental figure, episodes of brain tissue hypoxia (<20 mmHg) were most common during the first 3 days of ICU stay (in contrast to neuroglucopenia). There was no relation between neuroglucopenia and ptiO2 levels (p=0.306) and no change of ptiO2 during liberalization events (p=0.761). These data indicate that neuroglucopenia occurs independent of ptiO2 levels or brain tissue hypoxia, respectively.

Supplemental figure

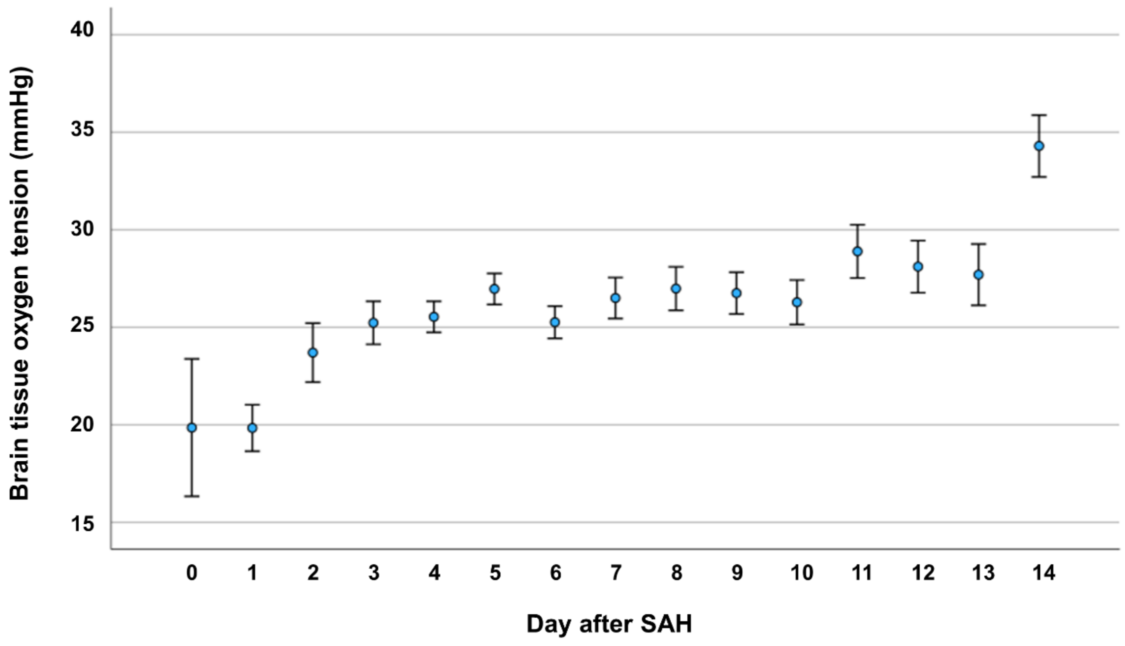

The supplemental figure shows the temporal course of mean brain tissue oxygen tension levels over the study period. Error bars represent the 95% confidence interval of mean. SAH = subarachnoid hemorrhage;
